# A comparison of licensed and un-licensed artisanal and small-scale gold miners (ASGM) in terms of socio-demographics, work profiles, and injury rates

**DOI:** 10.1186/s12889-017-4876-5

**Published:** 2017-11-06

**Authors:** Benedict N. L. Calys-Tagoe, Edith Clarke, Thomas Robins, Niladri Basu

**Affiliations:** 1 Department of Community Health, University of Ghana School of Public Health, Accra, Ghana; 2 Occupational and Environmental Health Unit, Ghana Health Service, Accra, Ghana; 3 Department of Environmental Health Sciences, University of Michigan School Of Public Health, Ann Arbor, MI 48109 USA; 4 Faculty of Agricultural and Environmental Sciences, McGill University, Montreal, QC H9X 3V9 Canada

**Keywords:** Miners, Occupational health, Occupational injuries, Public health, Workplace, Vulnerable populations, Informal sector

## Abstract

**Background:**

Artisanal and small-scale gold mining (ASGM) represents one of the most hazardous work environments. While formalization of this sector has been suggested (e.g., Minamata Convention) as a means to improve working conditions, we are unaware of empirical evidence that supports this notion.

This study aimed to compare sociodemographic profiles, work profiles, and injury rates among miners working in licensed versus un-licensed ASGM sites.

**Methods:**

In the Tarkwa mining region of Ghana, 404 small-scale miners were recruited in 2014 and interviewed regarding their occupational injury experiences over the preceding 10 years. Workers were drawn from 9 mining sites, of which 5 were licensed and 4 were not licensed.

**Results:**

Sociodemographic characteristics of miners from the two groups were relatively similar. Those currently working in an un-licensed mine have spent more time in the ASGM sector than those currently working in a licensed mine (94 vs. 70 months). Miners working in an un-licensed site tended to experience more injury episodes (e.g., 26% vs. 8% had 3 or more injury events) and not use personal protective equipment during the time of an injury (92% indicated to not using vs. 73%) when compared to miners working in a licensed site. A total of 121 injury episodes were recorded for 2245 person years of ASGM work. The injury rate for those working in un-licensed mines was 5.9 per 100 person years (59 injuries in 995 person years) versus 5.0 (62 injuries in 1250 person-years) in the licensed mines. When focusing on the male miners, there was a significant difference in injury rates between those working in a licensed mine (4.2 per 100 person years) versus an un-licensed mine (6.1 per 100 person years).

**Conclusions:**

These findings advance our understanding of injuries amongst ASGM workers, and help identify important differences in socio-demographics, work profiles, and injury rates between miners working in a licensed versus and un-licensed site. The findings suggest that certain working conditions in a licensed site may be safer.

## Background

Over the past decade artisanal and small-scale gold mining (ASGM) has proliferated worldwide due to powerful economic forces. Upwards of 15 million people may directly be involved in ASGM with another 100 million people estimated to be reliant upon the sector [1, 2]. Several health concerns exist in ASGM communities [3]. Much of the initial concern focused on human exposures to mercury as well as the poor health infrastructure within ASGM communities, although there is now greater awareness and evidence that a multitude of health hazards plague the ASGM sector including, for example, human exposures to many social stressors [4], toxic elements other than mercury [3], and noise pollution [5].

According to the Small-Scale Gold Mining law of Ghana (1989, PNDCL 218, section 21), small-scale (gold) mining is defined as “…mining (gold) by any method not involving substantial expenditure by an individual or group of persons not exceeding nine in number or by a co-operative society made up of ten or more persons”. When this is done using rudimentary tools such as shovels and pick axes, it is referred to as artisanal mining. Artisanal and small-scale gold mining, like other forms of mining, represents one of the most hazardous work environments [6]. For the ASGM sector, there is limited research addressing occupational hazards, though it is widely observed that miners are regularly injured because of falls, being struck by objects, exposure to extreme temperatures, misuse of or faulty power tools and equipment, and lacerations, as well as tunnel collapses resulting from weak ore formations or inadequate trenching and shoring. Deaths and injuries have been reported from several sites across Latin America, Asia, and Africa [7–9]. Few ASGM miners use personal protective equipment [10, 11], though the empirical evidence base for this as well as the preceding statements is relatively weak.

As the ASGM sector grows worldwide there is a pressing need for occupational hazards and injury factors to be quantitatively and rigorously studied so that findings may inform actions to improve working conditions. Furthermore, within the international UN Minamata Convention on Mercury there exists special mention of the ASGM sector (i.e., Article 7 and Annex C), and in particular a requirement of countries with ASGM activities that are more than insignificant to develop and implement a national action plan that includes public health strategies to protect vulnerable populations. One way forward, as mentioned in several government, NGO [11], and academic publications [12–14], as well as the Minamata Convention (Annex C 1-c) is to take steps to formalize the ASGM sector. It is believed that informal mining poses even more hazards than what may be found in a highly organized and/or regulated and/or large-scale operation. For example, the International Labour Organization has estimated that non-fatal accidents may be 7 times more common in mining operations that are informal when compared to large-scale operations [11]. The formalization process in Ghana requires the acquisition of land, notification of the Minerals commission, environmental impact assessment, and the payment of stipulated fees. While it is believed that formalization of the sector will help improve working conditions within ASGM sites, we are not aware of any empirical support of this notion. With this in mind, the objective of the current study was to compare socio-demographic profiles, work profiles, and injury rates among miners working in licensed versus un-licensed ASGM sites. We addressed this objective through further analysis of data we had previously collected in a cross-sectional study conducted in the Tarkwa region of Ghana in which we characterized the socio-demographics of ASGM miners as well as their work activities and work-related injuries [9].

## Methods

A cross sectional survey was carried out in Ghana’s Western Region between March and April of 2014 as previously detailed [9]. Briefly, 404 miners were recruited from 9 ASGM sites, 5 of which were licensed. The licensed mines were selected by simple random selection (from a list maintained by the district office of the Minerals Commission) of active mines that were licensed to operate within the study area. The unlicensed mines were identified using the concept of creating “contact zones”. This concept is often used in the context of highly asymmetrical relations of domination and subordination, especially among people with unusual power relations. Small-scale miners in Ghana are looked upon as “threats” or “a menace” to the environment and society, and their activities are usually clamped down by the security agencies. These miners are therefore very suspicious and unwilling to cooperate with “strangers”, including researchers, hence the use of this approach. The details are as elaborated by Calys-Tagoe et al. [9].

A structured, interviewer-administered questionnaire was used as previously described [9] to obtain information on socio-demographic characteristics, mining work history, and injuries. The injuries were categorized into mild (“no day lost” up to three days of absence from work), moderate (4–14 days of absence), and severe (absence for more than 14 days). The interviews were conducted by local trained medical staff proficient in English and Twi (the local dialect). The collected data was stored and analyzed electronically in SPSS (version 22), and double-keyed to ensure accuracy. Preliminary data analysis included tabulation of descriptive statistics for all measurements to understand the basic features of the dataset. Differences between licensed and un-licensed miners for key study variables were assessed using Chi-square and ANOVAs.

## Results

### Socio-demographics

A total of 404 ASGM workers from the Tarkwa mining district were interviewed (Table 1). These workers were drawn from 9 mining sites, of which 5 were licensed and 4 were not licensed. There were some variations in socio-demographic characteristics between the two groups. Notably, there were significantly more females sampled from the licensed sites; of the 32 females, only 2 worked in an un-licensed site. Nearly 75% of the miners were less than 40 years of age, with a mean age of 34 (range: 17–72 years). Close to 30% of the miners had reported having completed senior high school, and nearly two-thirds reported to living currently with a partner; none of these varied significantly between miners working in a licensed versus un-licensed site. Self-reported tobacco use (not shown in Table 1) was minimal with only 3.5% who indicated that they currently smoked tobacco; almost 90% indicated to having never smoked tobacco. Among the 44% who reported drinking alcohol within the past 12 months, 60% reported consuming an average of one drink per day. There were no significant differences in tobacco or alcohol use between licensed and un-licensed miners.

**Table 1:** Socio-demographic and work characteristics of the study population according to mine license status

Mine license status	Mine ID number	Sample population	Age (years)			Male (%)	High school completed (%)	Living with partners (%)	Total # months ever worked in ASGM			Total # months worked in current ASGM site		
			Mean	SD	Range				Mean (SD)	Median	Range	Mean (SD)	Median	Range
Licensed Mines	1	33	34.1	13.4	17–72	100	33.3	63.6	69 (83)	36	1–288	47 (59)	24	1–240
	2	63	34.8	10.0	19–60	79.4	22.2	58.7	66 (75)	36	1–360	37 (47)	17	1–180
	3	65	37.2	11.4	20–65	75.4	18.4	76.9	83 (80)	48	1–336	54 (58)	36	1–240
	4	84	30.9	8.8	18–55	100	23.8	63.1	67 (70)	51	1–360	41 (43)	24	1–240
	5	49	33.7	11.5	19–66	98	44.9	75.5	60 (70)	24	1–276	48 (58)	24	1–180
	Sub-Total	295	34.0	10.8	17–72	89.8	26.8	67.5	70 (75)	42	1–360	45 (53)	24	1–240
Un-licensed Mines	6	19	24.5	8.5	17–47	100	31.6	26.4	49 (74)	24	1–300	24 (32)	12	1–132
	7	16	32.6	9.3	22–58	100	43.8	37.5	70 (54)	48	4–168	59 (52)	36	4–168
	8	19	34.0	10.4	17–54	100	36.9	73.7	92 (63)	78	1–216	52 (59)	24	1–180
	9	56	36.3	8.9	22–57	96.4	28.6	76.7	119 (102)	120	1–360	83 (95)	24	1–288
	Sub-Total	109	33.2	10.0	17–58	98.2	33.1	61.4	94 (89)	60	1–360	63 (78)	24	1–288
Combined		404	33.8	10.6	17–72	92.1	28.5	65.8	76 (80)	48	1–360	50 (61)	24	1–288

### Work profiles

Participants self-reported to have worked in the ASGM sector from 1 month to 30 years, with a mean work duration of 76.3 months or 6.4 years (Table 1). Those currently working in an un-licensed mine had been working much longer in the ASGM sector than those currently working in a licensed mine (94 vs. 70 months or 7.8 versus 5.8 years on average). Further, 41% of miners currently employed in an un-licensed mine have worked in the ASGM sector for more than 10 years versus 22.4% of those currently employed in a licensed mine. The average number of years working at the individual’s current mine was 4.2, with the median number of years worked being 4, and the 25th and 75th percentiles being 1.1 and 10 years, respectively. Two hundred and fifty two (62.4%) of the miners had worked only at their current workplace (193 and 59 for licensed and unlicensed, respectively) throughout their mining career. Of the remaining 152 who have worked at two or more ASGM sites, 149 (98%) had worked in both licensed and unlicensed sites.

All participants reported involvement in one or more of the 7 key ASGM activities we queried, namely excavation (49%), crushing and grinding (46%), sifting and shanking (13%), washing and sluicing (39%), amalgamation (33%), burning (38%), and carrying loads (6%). More than 50% of the respondents indicated to being routinely involved in more than one activity, with 25% of them indicating to be involved in 4 or more activities on a regular basis. While there were no statistically significant differences between the licensed and un-licensed miners in terms of the number of work activities that they reported being currently involved with, the proportion of un-licensed miners involved in amalgamation (43% vs. 29%) and burning (54% vs. 32%) was higher than that for the licensed miners.

### Injury rates

Over the last 10 years (which is the time period for which participants were specifically queried about experiencing injuries from all their ASGM-related activities), the 404 study participants worked a total of 2245 person years in the ASGM sector (1250 years in licensed mines and 995 years in un-licensed mines). During this capture period, 95 of the 404 individuals interviewed reported at least one injury event that caused them to miss days of work or hampered their ability to work effectively when they showed up to work. The overall incidence proportion of injury was 23.5%, with 17.3% for those working in a licensed mine and 40.3% for those working in an un-licensed mine. The majority of respondents who self-reported an injury event experienced a single injury (75/95). The number of miners experiencing 2, 3, 4, and 5 injuries over the 10-year period was 13, 5, 0, and 1, respectively, for a total of 121 injury episodes.

We further analyzed the injury events according to key variables (Table 2). Even though miners moved between licensed and unlicensed sites, our survey was designed to link a particular injury event with the mine’s registration status at the time of injury. Miners working in an un-licensed site tended to experience more injury episodes (e.g., 26% vs. 8% had 3 or more injury events) and not use personal protective equipment during the time of an injury (92% indicated to not using vs. 73%) when compared to miners working in a licensed site. For other variables of interest (e.g., activity at the time of injury, time of day when the injury occurred, cause of injury, and severity of injury), there were no statistically significant differences between those working in a licensed versus un-licensed mine. However, some general observations warrant mention. First, the incidence of injury was most associated with two activities (excavation and crushing), which accounted for 82% of all injury-related events. Second, injuries tended to occur more often in the morning than during later parts of the day though we did not characterize frequency of activities throughout the entire work day. Third, the cause of injury in about two-third of all incidents was reported being struck by an object, though there were more diverse causes reported by miners from the un-licensed sites (e.g., fires, fall from height, and physical assault). Fourth, while a higher proportion of injuries amongst licensed miners were reported to be severe this was not to a level of statistical significance.

**Table 2:** Attributes of self-reported injury related events comparing those working in a licensed versus un-licensed ASGM site

Injury-related events	Licensed (*N* = 62) %	Unlicensed (*N* = 59) %	All sites (*N* = 121) %	*p*-value
Activity at the time of injury				
Excavation	55	63	59	0.16
Crushing	23	24	23	
Washing/sluicing	3	0	2	
Burning	0	3	2	
Movement between locations	19	9	14	
Time of day injury occurred				
Morning	44	44	44	0.79
Afternoon	39	34	36	
Evening/night	18	22	20	
Cause of injury				
Struck by an object	76	58	67	0.10
Machinery/tool	11	15	13	
Fire/flames/heat	0	3	2	
Fall from a height	0	7	3	
Fall on level ground	5	0	3	
Physical assault	0	3	2	
Other	5	9	7	
Severity of injury				
Mild	32	29	31	0.20
Moderate	40	29	35	
Severe	27	42	35	
Number of injury episodes experienced by individual miners				
1	71	56	64	**0.04**
2	21	19	20	
3	8	17	12	
4	0	0	0	
5	0	9	4	
Work experience (in years)				
0–5	52	37	45	**0.02**
6–10	26	20	23	
11–15	5	19	12	
16–20	10	22	16	
> 20	8	2	5	
Training				
Yes	34	24	29	0.22
No	66	76	71	
Use of PPE at the time of injury				
Yes	27	9	18	**0.009**
No	73	92	82	

Values in the cells represent column percentages. Significance was tested using chi-squared tests. *N* = sample size (bold numbers)

With a total of 121 injury episodes and 2245 person years of ASGM work, the overall injury rate was calculated at 5.39 per 100 person years (Table 3). The injury rate for those working in un-licensed mines was 5.93 per 100 person years (59 injuries in 995 person years) versus 4.96 (62 injuries in 1250 person-years) in the licensed mines resulting in a risk ratio of 1.2. Given that most women worked in a licensed site, and that we previously documented them to have a greater injury rate than men [9], we further sub-divided the analyses as outlined in Table 3. When focusing strictly on the male miners, the injury rate was significantly different between those working in a licensed mine (4.16 per 100 person years) versus an un-licensed mine (6.05 per 100 person years). Similar analyses were performed by strictly looking at injury events classified as severe (i.e., work absence for more than 14 days), and this mainly revealed that the rate of such events were more common in licensed mines (Table 4).

**Table 3:** A comparison of injury rates between sexes and mine licensing status

	Overall injury rate	Injury rates at unlicensed sites	Injury rates at licensed sites	Rate ratio (95% CI)	*P*-value
Both sexes	121 injuries / 2245 person years =5.39 injuries/100 person years	59 injuries / 995 person years =5.93 injuries/100 person years	62 injuries / 1250 person years =4.96 injuries/100 person years	1.20 (0.83–1.71)	0.33
Males	107 injuries / 2128 person years =5.03 injuries/100 person years	59 injuries / 975 person years =6.05 injuries/100 person years	48 injuries / 1153 person years =4.16 injuries/100 person years	1.45 (0.99–2.14)	0.05
Females	14 injuries / 117 person years =11.97 injuries/100 person years	0 injuries / 20 person years =0 injuries/100 person years	14 injuries / 97 person years =14.43 injuries/100 person years	/	< 0.01
Rate Ratio	0.42 (0.25–0.76)	/	0.29 (0.16–0.54)		
*P*-value	< 0.01	0.30	< 0.01		

The *p*-values refer to rate comparisons within a particular column or row, and for the rate ratio the 95% confidence interval is provided in the brackets

**Table 4:** A comparison of injury rates deemed to be ‘severe’ between sexes and mine licensing status

	Overall injury rates deemed “severe”	Severe injury rates at unlicensed sites	Severe injury rates at licensed sites	Rate ratio (95% CI)	*P*-value
Both sexes	42 injuries / 2245 person years =1.9 injuries/100 person years	14 injuries / 995 person years =1.4 injuries/100 person years	28 injuries / 1250 person years =2.2 injuries/100 person years	0.64 (0.32–1.18)	0.15
Males	41 injuries / 2128 person years =1.9 injuries/100 person years	14 injuries / 975 person years =1.4 injuries/100 person years	27 injuries / 1153 person years =2.3 injuries/100 person years	0.61 (0.31–1.16)	0.14
Females	1 injuries / 117 person years =0.9 injuries/100 person years	0 injuries / 20 person years =0 injuries/100 person years	1 injuries / 97 person years =1.0 injuries/100 person years	/	0.82
Rate Ratio		/	2.3 (0.43–47)		
*P*-value		0.75	0.45		

The *p*-values refer to rate comparisons within a particular column or row, and for the rate ratio the 95% confidence interval is provided in the brackets

We also explored the injury rates for two variables from Table 2 that were statistically significant, namely number of injury episodes and work experience. The injury rates were not different between unlicensed and licensed miners who experienced 1 injury event (14.35/100 yrs. vs. 18.91/100 yrs., respectively; *p* = 0.11) or 2 or more events (34.62/100 yrs. vs. 43.59/100 yrs., respectively; *p* = 0.23). Injury rates varied according to work experience in both the unlicensed and licensed miners with rates being significantly higher in the less experienced miners (Table 5). Furthermore, among the miners with more than 5 years of work experience, the injury rates were more than 50% higher in the unlicensed group.

**Table 5:** A comparison of injury rates between mine licensing status and the work experience of the miners

	Overall injury rate	Injury rates at unlicensed sites	Injury rates at licensed sites	Rate ratio (95% CI)	*P*-value
Both Groups	121 injuries / 2245 person years =5.39 injuries/100 person years	59 injuries / 995 person years =5.93 injuries/100 person years	62 injuries / 1250 person years =4.96 injuries/100 person years	1.20 (0.83–1.71)	0.33
5 years and less of ASGM work experience	54 injuries / 659 person years =8.19 injuries/100 person years	20 injuries / 245 person years =8.16 injuries/100 person years	34 injuries / 414 person years =8.21 injuries/100 person years	0.99 (0.57–1.73)	0.99
More than 5 years of ASGM work experience	67 injuries / 1586 person years =4.22 injuries/100 person years	39 injuries / 750 person years =5.20 injuries/100 person years	28 injuries / 836 person years =3.35 injuries/100 person years	1.53 (0.96–2.52)	0.03
Rate Ratio	1.94 (1.36–2.78)	1.57 (0.92–2.69)	2.45 (1.49–4.04)		
*P*-value	< 0.001	0.05	< 0.001		

The *p*-values refer to rate comparisons within a particular column or row, and for the rate ratio the 95% confidence interval is provided in the brackets

## Discussion

There is growing concern worldwide about the ASGM sector, and in particular the occupational health risks faced by ASGM workers. One strategy forward, as articulated in academic papers [12–14] as well as in the UN Minamata Convention (Annex C 1-c), is to take steps to formalize the sector. For example, a polling exercise that involved diverse ASGM stakeholders and experts from across Ghanaian institutions revealed that the promotion of conditions to help ASGM miners register, regularize, and develop their mining activities consistently scored amongst the most preferred option for improving the health, environmental, and socioeconomic problems faced by ASGM communities [13]. While there are widely held beliefs and anecdotes that unlicensed mining poses more occupational hazards than what may be found in a regulated or large-scale operation [11], to our knowledge there has been no empirical evidence generated in support of this notion. We believe the current study is the first to scientifically compare a group of licensed and un-licensed ASGM mine workers. In doing so, we are able to compare the groups in terms of their socio-demographics, ASGM work profiles, and injury rates.

In Ghana, like other countries, those who engage in illegal ASGM activities are often vilified in the media and by governments [15] leading towards a simple perception that these two groups are different. In the current study we found no striking socio-demographic differences (e.g., age, education level, living status, alcohol and cigarette consumption) between the miners currently working in a licensed and un-licensed site. Moreover, the fact that 98% of those miners who have worked at more than one site, have worked in both licensed and un-licensed sites at some point in time suggests that, at least in Ghana, there are not substantial or consistent differences with respect to the types (socioeconomic backgrounds, etc.) of individuals who may be found working in licensed vs. unlicensed mines. This also suggests that differences between licensed and unlicensed miners in terms of work-related safety (e.g., acute injuries) is most likely due to the differences in levels and types of hazards faced in the different types of mines, rather than any preexisting socioeconomic differences in the individuals employed (one exception from our work was related to work experience, and is discussed below). While there was a significant difference in terms of the number of females employed, this may likely not a true representation of the overall situation owing to the relatively small sample size. Here, only two women were sampled from the un-licensed ASGM sites and they reported zero injuries. The overall injury rate for females (11.9 injuries per 100 person years) is much greater than that of the men, and something that we previously discussed [9]. The role of females within the ASGM sector is important, discussed elsewhere by us and others [1, 16, 17], and warrants much more attention.

There is some difficulty in generalizing whether an ASGM miner exclusively operates in an illegal manner, and this represents an important limitation of the current study (and likely also other studies concerning ASGM). In the current study, 65 and 54% of miners working in a licensed and unlicensed site currently had worked only in that current site throughout their mining career. Of the remaining miners who have worked at two or more ASGM sites, 98% indicated to have worked in both licensed and unlicensed sites. As such, there is a tendency of many miners to move between licensed and unlicensed operations. In conversations we had with many study participants, they indicated being motivated mostly by financial gains and that they did not discriminate between the various mining sites based on their registration status, but rather on which of them had the ability to meet their financial demands. It should also be noted that ASGM miners in Ghana themselves are not licensed but rather the site in which they are operating.

Further comparisons of work characteristics between ASGM miners working in a licensed and unlicensed operation were revealing. Those who currently work in an un-licensed mine have been working longer in the ASGM sector. In terms of specific ASGM activities we queried (key ones being excavation, crushing and grinding, sifting and shanking, washing and sluicing, amalgamation, burning, and carrying loads) we found no difference between the groups. More than half of the respondents in each group indicated to being routinely involved in more than one activity. Although of potential interest, the proportion of un-licensed miners involved in amalgamation and burning was higher than the licensed miners. This could bode problems, in particular, for mercury exposure which others have shown to be largely due to burning activities [10]. Even though few miners are involved in burning mercury, the released chemical contaminates the entire worksite and broader community thus rendering everyone potentially exposed.

One of the key findings of the current study was the difference between the licensed and un-licensed miners with respect to their injury rates. The incidence proportion of injury was 17.3% for those working in a licensed mine versus 40.3% for those working in an un-licensed mine. Focusing strictly on the male miners, the injury rate among un-licensed miners (6.1 injuries per 100 person years) was significantly higher than licensed miners (4.2 injuries per 100 person years). In addition, among the miners with more than 5 years of work experience, the injury rates were more than 50% higher in the unlicensed group (5.2 injuries per 100 person years) than the licensed group (3.4 injuries per 100 person years). Comparing these values to other studies has proven challenging owing to the lack of information available. We are unaware of any studies comparing injury profiles (or other occupational and health measures) between licensed and unlicensed miners. Existing occupational health studies concerning ASGM are quite limited; they are largely descriptive, and vary greatly in methodology making it difficult to generalize and make comparisons. For example a previous study of small-scale miners in the Democratic Republic of the Congo [18] calculated an injury rate of 392 accidents per 100 person years though methodological differences (e.g., their sampling frame was one year and extended beyond ASGM) make it difficult to properly compare.

In addition to the injury rate we found that miners working in an un-licensed site tended to experience more injury episodes when compared to miners working in a licensed site though when stratified (1 injury event versus 2 or more) and normalized for person-years, we calculated no significant differences. The miners working in an un-licensed site also reported using less personal protective equipment during the time of the injury. A study from Ghana’s north found that the majority of ASGM miners do not use personal protective equipment including items such as hardhats, gloves, and steel toed boots [10]. Here we extend upon this study and report that the use of personal protective equipment may be less for those working in an un-licensed operation. Though, a limitation with our work is that we only queried about the use of personal protective equipment when an injury was self-reported, and so we do not have data on all the 404 miners that we engaged with. This would be an important area of future inquiry.

This study has several strengths. Foremost, to our knowledge it is the first epidemiological study to compare variables between miners who work in licensed and un-licensed ASGM sites. The sample size is relatively robust when compared to other ASGM studies, and the sampling strategy involving multiple sites was aimed at reducing bias. Nonetheless there are important limitations of our study that warrant mention. Recall bias is an inherent limitation in this type of study even though we utilized validated survey instruments and employed trained field staff, and also do not feel that those working in an un-licensed mine would recall past events differently than those working in a licensed mine. The sampling design was aimed at recruiting workers from both licensed and unlicensed sites. While it has been estimated that upwards of 85% of Ghana’s ASGM workers do not have licenses [19], we were unable to properly enumerate this in the study region though we have no reason to believe that the selected mines were atypical.

## Conclusion

In conclusion, these findings advance our understanding of injuries amongst ASGM workers, and help identify important differences in socio-demographics, work profiles, and injury rates between miners working in a licensed versus and un-licensed site. Such findings are important given that a number of authorities, including Annex C 1-c of the UN Minamata Convention, indicate formalization to be a potential solution to helping improve the health, environmental, and socioeconomic problems faced by ASGM communities though to our knowledge there has been no empirical evidence in support of this notion and thus our study fills an important knowledge gap.
